# CT-Guided Transthoracic Biopsy of Pulmonary Lesions: Diagnostic versus Nondiagnostic Results

**DOI:** 10.3390/diagnostics12020359

**Published:** 2022-01-31

**Authors:** Cristina Borelli, Doriana Vergara, Anna Simeone, Luca Pazienza, Giulia Castorani, Paolo Graziano, Concetta Di Micco, Carla Maria Irene Quarato, Marco Sperandeo

**Affiliations:** 1 Unit of Radiology, IRCCS Casa Sollievo della Sofferenza, 71013 San Giovanni Rotondo, FG, Italy; dorianavergara@hotmail.it (D.V.); a.simeone@operapadrepio.it (A.S.); lucapazienza@libero.it (L.P.); giulia.castorani90@gmail.com (G.C.); 2 Unit of Patology, IRCCS Casa Sollievo della Sofferenza, 71013 San Giovanni Rotondo, FG, Italy; p.graziano@operapadrepio.it; 3 Unit of Oncology, IRCCS Fondazione Casa Sollievo della Sofferenza, 71013 San Giovanni Rotondo, FG, Italy; doctor.dimicco@gmail.com; 4 Institute of Respiratory Diseases, Policlinico “Riuniti” di Foggia, 71122 Foggia, FG, Italy; carlamariairene.quarato@gmail.com; 5 Unit of Interventional and Diagnostic Ultrasound of Internal Medicine, IRCCS Fondazione Casa Sollievo della Sofferenza, 71013 San Giovanni Rotondo, FG, Italy; sperandeomar@gmail.com

**Keywords:** computed tomography, transthoracic needle biopsy, pulmonary lesions, lung cancer, nondiagnostic results

## Abstract

(1) Background: Despite the high accuracy of CT-guided transthoracic biopsy for diagnosis of pulmonary lesions, in a certain amount of cases biopsy results may indicate the presence of nonspecific findings or insufficient material. We aimed to investigate the effectiveness of CT-guided transthoracic biopsy of pulmonary lesions in providing a specific diagnosis and to analyze the variables affecting biopsy results. (2) Methods: In this retrospective study, a total of 170 patients undergoing 183 CT-guided transthoracic biopsies of pulmonary lesions were included. The clinical, radiological and pathological data were reviewed to classify biopsy results as diagnostic or nondiagnostic and to identify which variables were associated with the two groups. (3) Results: The biopsy results were diagnostic in 150 cases (82.0%), of which 131 (87.3%) positive for malignancy and 19 (12.7%) with specific benign lesions, and nondiagnostic in 33 cases (18.0%). Twenty-two of the thirty-three (66.7%) nondiagnostic cases were finally determined as malignancies and eleven (33.3%) as benign lesions. In the diagnostic group, all the 131 biopsies positive for malignancy were confirmed to be malignant at final diagnosis (87.3%); of 19 biopsies with specific benign lesions, 13 cases were confirmed to be benign (8.7%), whereas six cases had a final diagnosis of malignancy (4%). Multivariate analysis showed increased risk of nondiagnostic biopsy for lesions ≤ 20 mm (*p* = 0.006) and lesions with final diagnosis of benignity (*p* = 0.001). (4) Conclusions: CT-guided transthoracic lung biopsy is an effective technique for the specific diagnosis of pulmonary lesions, with a relatively acceptable proportion of nondiagnostic cases. Small lesion size and final benign diagnosis are risk factors for nondiagnostic biopsy results.

## 1. Introduction

Computerized tomography (CT)-guided transthoracic lung biopsy is a well-established and commonly performed procedure in thoracic interventional radiology for tissue diagnosis of pulmonary lesions. The technique is minimally invasive and safe, with acceptable low complication rates [1,2,3,4].

Indications of transthoracic lung biopsy include indeterminate pulmonary nodules or mass lesions not subjectable to transbronchial biopsies, multiple nodules in patients with unknown extra-thoracic malignancy, perihilar mass lesions after failed or negative bronchoscopy, persistent focal infiltrates for which no diagnosis is possible with other investigations [5]. In addition, transthoracic lung biopsy is often integrated into medical work-up of lung cancer patients. Tissue sampling allows for diagnostic confirmation of lung cancer, exclusion of secondary neoplasms and assessment of histological and molecular subtypes [6].

In particular, since the introduction of specific chemotherapy drugs with different effectiveness based on lung cancer histotype and the approval of targeted and immunotherapeutic agents, lung biopsy has gained great importance in histological and molecular characterization of non-small cell lung cancer (NSCLC) [7,8]. In this contemporary context of personalized therapy, tumor subtyping and molecular analysis have become essential in NSCLC, in order to identify specific tumor characteristics and enable more individualized treatments [9].

Many previous studies on transthoracic lung biopsy have focused on the evaluation of its diagnostic yield, reporting high accuracy rates, ranging from 79,7% to 96,9%, and overall sensitivity and specificity for the diagnosis of lung cancer of 90% and 97%, respectively [1,10,11,12,13]. Furthermore, various reports have analyzed factors thought to influence the diagnostic accuracy of the procedure. Some authors suggested that lesions with a diameter of 20 mm or smaller constitute a risk factor for diagnostic failure, while others found that lesions larger than 50 mm are likely to have diagnostic failure [10,14,15,16]. Instead, a final diagnosis of malignancy, clinical suspicion of primary lung cancer, superior lobe location of the lesion and short needle path length (≤40 mm) have been demonstrated to be significant factors for diagnostic success [15,17,18].

Despite the excellent sensitivity and specificity of transthoracic lung biopsy in diagnosis of pulmonary malignancies, with a rate of false positive of only 1–2% [13], in clinical practice, a certain amount of biopsies that do not give a specific diagnosis remains undetermined. These biopsies are those with no disease diagnosed or with insufficient material collected and are commonly considered as “nondiagnostic”. Instead, lung biopsies with a specific diagnosis, indicating a malignant or a benign lesion, are commonly considered “diagnostic” [19,20]. When a specific diagnosis is established at biopsy, the result, if concordant with clinical and radiological data, is usually considered conclusive. Nondiagnostic biopsy results have an outcome more uncertain and the clinical decision of observing the lesion or proceeding with further invasive investigations may be challenging. Indeed, in a recent study, Fontaine-Delaruelle et al. [21] have examined final diagnoses of nondiagnostic biopsy specimens demonstrating that one half of them were falsely negative for malignancy.

Knowledge of the characteristics and the final outcomes of diagnostic or nondiagnostic biopsies may provide valuable information to guide management decisions, mainly of lesions with uncertain diagnosis at biopsy.

So far, only a few studies have focused on the dichotomy between diagnostic and nondiagnostic results of CT-guided lung biopsy [19,20].

Thus, in the present study we analyzed diagnostic and nondiagnostic results from CT-guided biopsy of pulmonary lesions in order to investigate the effectiveness of the lung biopsy in providing a specific diagnosis of malignancy or benign disease, and to identify the factors affecting biopsy results.

## 2. Materials and Methods

### 2.1. Patients

This study was approved by the institutional research ethics committee (TACE-CSS, n. 106/2018).

All consecutive CT-guided lung biopsies recorded in our database from January 2014 to December 2020 were retrospectively analyzed. A total of 235 procedures were assessed. Inclusion criteria were patients who underwent CT-guided transthoracic biopsy of pulmonary lesions with available clinical, radiological and pathological data. Fifteen canceled biopsy procedures were excluded from the analysis. Various factors contraindicated lung biopsies such as the inability of the patient to cooperate (*n* =4), too small size or disappearance of the lesion at pre-procedural CT scan (*n* = 2), lack of a safe pathway to the lesion (*n* = 1), patients’ refusal (*n* = 5) and inadequate coagulation status (*n* = 2). In 11 cases, CT-guided transthoracic procedures were performed for preoperative methylene blue localization of non-palpable pulmonary nodules and in 26 cases data regarding the final outcome of the lesions were not available. After excluding these cases, the final dataset consisted of 183 CT-guided lung biopsies performed in 170 patients.

Bronchoscopy or transbronchial biopsies were nondiagnostic or not feasible in enrolled patients. The biopsy procedure was repeated in 13 patients due to clinical requirements.

Before proceeding to biopsy, bleeding time parameters were measured in all patients in order to exclude a potential uncorrectable coagulopathy (international normalized ratio ≥1.5, platelet count < 50,000/mcL).

Any platelet antiaggregant and anticoagulant treatment was suspended a few days prior to the procedure, in accordance with the published guidelines on perioperative anticoagulation [22].

All patients received verbal explanations about the biopsy procedure and its possible complications. Afterward, they read and signed the written informed consent for the biopsy.

### 2.2. Biopsy Procedure

All the procedures were performed by the same radiologist having more than 20 years of experience in thoracic radiology and CT-guided biopsies, under the guidance of a 16-rows CT scanner (Aquilion, Toshiba Medical System, Tokyo, Japan). A semiautomatic modified Menghini-type needle (Biomol, HS Medical, Rome, Italy), with a caliber of 18 or 21 gauge, was used. This biopsy device is equipped with a spring mechanism, that helps the charge and the automatic release of the syringe plunger, simplifying the procedure and leaving to the physician sampling control.

The patient was positioned on the basis of the previous diagnostic CT examination, if present. A pre-procedural chest CT scan was performed (1 mm of thickness) in order to exactly localize the pulmonary lesion and to find the more suitable site of percutaneous access. Subsequently, after positioning a radiopaque cutaneous marker, a new CT scan was performed on the site of interest (5 cm cranial and caudal to the lesion). A biopsy route was chosen taking care to avoid the fissures, bullae, large vessels, visible bronchi, ribs, and scapulae. Generally, the needle was introduced as perpendicular to the pleural surface as possible, but if the lesion was covered by a rib the access was performed through the upper or lower intercostal space with an oblique direction.

The patient was instructed to hold his breath during the CT scanning and the biopsy process.

**Figure 1:**
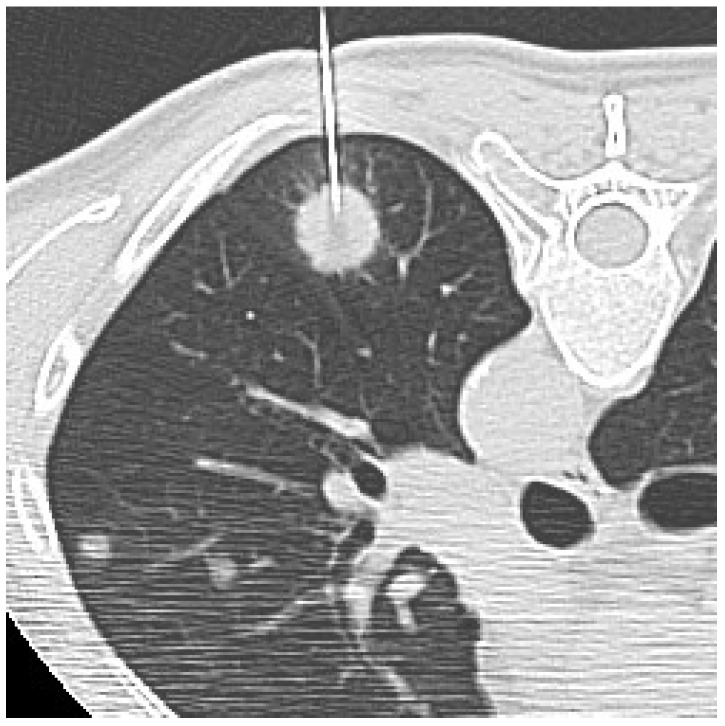
CT-guided biopsy of a solid pulmonary lesion in the left lower lobe. Supine unenhanced CT scan obtained before sampling shows a satisfactory position of the needle tip within the lesion.

After local anesthesia of the skin and subcutaneous tissues with 10–20 mL of carbocaine, the initial puncture was performed without penetrating the pleura. CT images were obtained to check the position of the needle tip. At this point, if the direction was appropriate, the needle was pushed forward to the lesion according to the planned trajectory route, otherwise, insertion angle or position was corrected. When the nodule was penetrated, the needle tip was again checked on CT (Figure 1). After CT confirmation of a satisfactory needle tip position, biopsy samples for both histological and cytological analysis were collected.

Since a pathologist was not routinely present during the procedure, further sampling was eventually performed by the operator on the basis of his visual inspection of material adequacy.

After biopsy, the resected specimens were put into previously prepared sterile 10% formalin solution for histological evaluation. The remnant material contained within the needle was immediately smeared on slides and sent to a pathologist for additional cytological analysis. Residual small tissue fragments were also collected from the needle and immersed in a preservative solution for the cell blocks.

At the end of the procedure, a CT scan with 1 mm of slice thickness (15 cm cranial and caudal to the lesion) was performed to detect intra-procedural complications such as pneumothorax and pulmonary parenchymal hemorrhage around the lesion and the puncture needle tract. Moreover, all patients underwent chest X-ray examination 6 h after biopsy, and late if necessary, to assess post-procedural complications. Asymptomatic patients with mild pneumothorax were treated conservatively. Chest tube drainage was considered in patients with symptomatic pneumothorax or in presence of large pneumothorax (greater than 30% of the volume of the affected lung). Post-biopsy hemoptysis was also monitored and eventually treated depending on the clinical status of the patient and the volume of blood loss.

### 2.3. Data Analysis

All medical charts, radiologic reports and intra-procedural/immediate post-procedural CT images as well as the subsequent chest X-rays were retrospectively evaluated.

Patient characteristics including age, sex, presence or absence of emphysema on CT images, smoking status and history of cancer were collected.

Lesion parameters such as size, CT attenuation, presence of spiculation and anatomic location were also recorded. Lesion size, defined as the longest diameter of the lesion, was measured at lung windows settings on axial CT images and, based on CT attenuation, lesions were categorized as solid or partially solid.

Data regarding biopsy technique consisted of indication to procedure, needle size, needle path length, number of samples and number of repeated biopsies for a single session. Needle path length was determined as distance from the pleura to target lesion according to needle trajectory.

Procedure-related complications included complications encountered during and up to 24 h from the biopsy, as noted on the radiologic report or directly on images. Complications were classified as minor or major according to the Society of Interventional Radiology (SIR) Guidelines [23]. Minor complications consisted of pneumothorax without need for intervention, small amount of pulmonary hemorrhage, and transient hemoptysis. Major complications consisted of pneumothorax requiring chest tube placement, hemothorax, hemoptysis requiring intervention, air embolism, needle tract seeding, and death.

On the basis of pathological reports, biopsy results were classified as diagnostic or nondiagnostic, according to previous studies [19,20]. Samples were considered diagnostic in presence of malignancy or specific benign results. Samples were defined nondiagnostic if the sample obtained was insufficient or showed normal lung and bronchial tissue, fibrosis, necrosis, nonspecific inflammation or atypical cells.

For malignant cases, diagnosis of lung cancer and tumor classification were made using morphological characteristics and, if necessary, immunohistochemistry. Based on these analyses, malignant lesions were further grouped by histological type.

Surgical pathology and post-procedural course of disease were considered as the standard of reference for the final diagnosis. In particular, based on clinical and radiological follow-up, the lesion was determined to be malignant in case of increasing size, regression after anticancer therapy, or occurrence of new metastases, and benign if it regressed spontaneously or with conservative medical treatment or remained stable. The mean duration of follow-up was 1 year.

If the patient underwent two biopsies for the same lesion, each biopsy was considered as a separate event and independently reviewed and categorized.

### 2.4. Statistical Analysis

All data were presented as mean values ± standard deviation for continuous variables, and as percentages for categorical variables.

The differences between diagnostic and nondiagnostic cases in terms of patient-, lesion-, and procedural-related variables were analyzed using Student’s t-test for continuous values and Pearson’s Chi-squared test or Fisher’s exact test for categorical values.

A *p* value of less than 0.05 was determined to be significant.

Subsequently, multivariate logistic regression analysis was performed to identify independent risk factors for biopsy failure intended as nondiagnostic pathological results. Factors with a *p* < 0.05 in univariate analyses were included in the multivariate analysis.

Statistical analysis was performed by using SPSS statistics 20.0 (IBM Business Analytic, NY, USA).

## 3. Results

### 3.1. Study Population Characteristics

The details about patients and lesions characteristics, biopsy technique and complications are provided in Table 1.

The 170 patients included in the study were 120 males and 50 females, with ages ranging from 15 to 89 years (mean age, 68.1 years). Thirteen patients underwent two biopsies for the same target lesion, therefore the dataset consisted of 183 biopsies. The repeat biopsies were performed for a previously nondiagnostic biopsy result (*n* = 8), for mutational analysis or subtype assessment (*n* = 3) if a certain diagnosis of malignancy was made, and for persistent suspicion of malignancy despite a diagnosis of the specific benign lesion (*n* = 2). In particular, the 8 patients with nondiagnostic biopsy results required a re-biopsy to rule out malignancy after inadequacy of previously sampling (*n* = 5) or nonspecific benign result at initial biopsy (*n* = 3).

Most of the lung lesions were located in the upper and middle lobes (59.6%) and showed a solid appearance at CT images (91.3%).

The mean lesion diameter was 35.9 mm, ranging from 11 to 91 mm.

Biopsy procedures were mainly performed for diagnosis and characterization of lung cancer, with histotype assessment and molecular analysis (65.6%), and used 18 gauge needles in 110 cases (60.1%) and 21 gauge needles in 73 cases (39.9%).

In our study, minor complications occurred in 66 biopsies, with 3 patients developing complications in two separate procedures, and major complications occurred in 3 biopsies (Table 1). In particular, 36 cases of pneumothorax were observed (19.7%). Only three cases (1.6%) required chest tube placement, with a complete resolution of the pneumothorax within 24–48 h. Tension pneumothorax or other major complications did not occur in any patient. There were 29 cases of parenchymal bleeding (15.9%) and 4 cases of hemoptysis (2.2%), but none of them required blood transfusion or any other treatment.

### 3.2. Biopsy Results

Of the 183 CT-guided lung biopsies included in the dataset, 150 (82.0%) and 33 (18.0%) biopsies showed diagnostic and nondiagnostic results, respectively.

The nondiagnostic biopsies contained nonspecific benign features such as normal structures, fibrous tissue, necrosis or inflammatory cells (*n* = 13; 39.4%), atypical cells (*n* = 6; 18.2%) or insufficient material for diagnosis because of cellular paucity (*n* = 14; 42.4%).

Twenty-two of the thirty-three (66.7%) nondiagnostic cases were proved to be malignant and eleven (33.3%) were proved to be benign.

All 6 cases with atypical cells at biopsy were finally diagnosed as malignant (100%). Among the remaining nondiagnostic cases, the final diagnosis of malignancy was made in 5 of 13 cases with nonspecific benign features (38.5%) and in 12 of 14 cases with inadequate specimens (85.7%).

In the 22 malignant cases with nondiagnostic biopsy results, the final diagnosis was achieved by repeat CT-guided biopsy in 5 cases (22.7%), surgery in 14 cases (63.6%) and clinical and radiological follow-up in 3 cases (13.7%). In the 11 benign cases with nondiagnostic biopsy results, the final diagnosis was made by re-biopsy in 2 cases (18.2%), surgery in 4 cases (36.4%) and clinical and radiological follow-up in 5 cases (45.4%). Seven of the 8 biopsies repeated after an initial nondiagnostic biopsy provided a diagnostic result, whereas in one case the repeat biopsy was again nondiagnostic and the final diagnosis of benignity was established on the basis of spontaneous lesion regression at radiological follow-up.

The 150 biopsies with diagnostic results were positive for malignancy in 131 cases (87.3%) and showed specific benign lesions in 19 cases (12.7%). Among the diagnostic biopsies, all the 131 cases with biopsy result of malignancy were confirmed to be malignant at final diagnosis (87.3%); there were no malignant biopsies ultimately diagnosed as benign. Thirteen diagnostic cases had a conclusive diagnosis of benignity concordant with the initial biopsy result (8.7%). Six cases with specific benign lesions at biopsy had a final diagnosis of malignancy (4%).

In the 150 cases with diagnostic biopsy, the final diagnosis of malignant or benign disease was determined by surgical resection in 52 cases (34.7%), pathological results of the biopsy in combination with laboratory findings and clinical course in 96 cases (64%), and re-biopsy in 2 cases (1.3%).

Considering all biopsies, the final diagnosis was malignancy in 159 cases (86.9%) and benign disease in 24 cases (13.1%).

**Figure 2:**
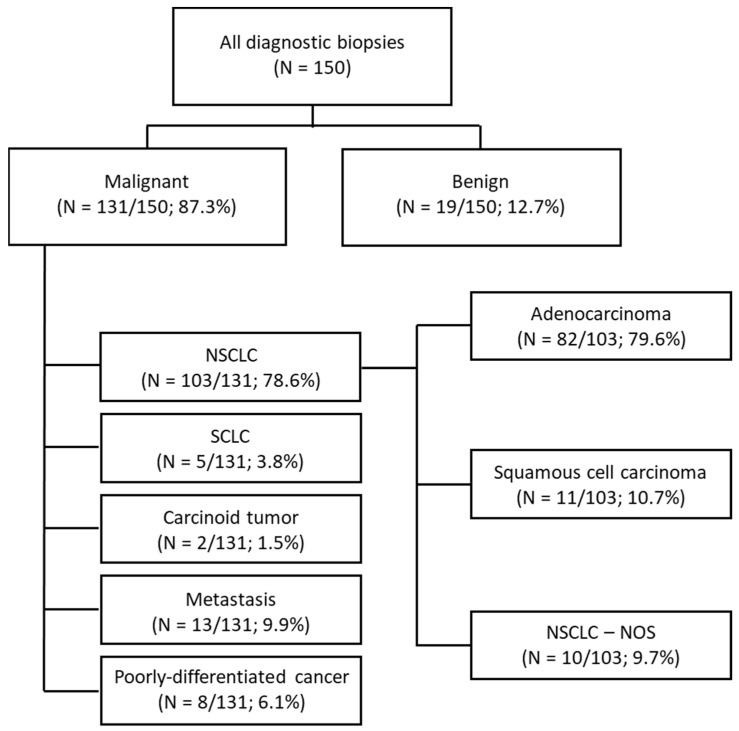
Flow diagram showing the distribution of diagnostic biopsy results of malignancy.

As reported above, the diagnostic biopsies were positive for malignancy in 131 cases, that included 103 NSCLC (78.6%), 5 small-cell lung cancer (3.8%), 2 carcinoid tumors (1.5%), 13 metastasis (9.9%) and 8 poorly-differentiated cancer of unknown primary (6.1%). Among the 103 cases with initial diagnosis of NSCLC, a total of 93 cases were specifically subtyped and classified in adenocarcinoma (*n*= 82; 79.6%) and squamous cell carcinoma (*n* = 11; 10.7%). Ten cases of NSCLC—not otherwise specified were reported (9.7%). The distribution of all biopsy results with diagnosis of malignancy is detailed in Figure 2.

Morphology alone or in combination with immunochemistry was used to discriminate metastases from primary lung cancer, small cell lung carcinoma from NSCLC, and, in this last group, adenocarcinoma from squamous cell carcinoma. Considering all 131 biopsies with a diagnosis of malignancy, in 89 cases tumor classification was possible by using morphological findings alone (67.9%) and in 24 cases with both morphological and immunohistochemical findings (18.3%); in 18 cases tumor remained unclassified (13.7%). In particular, the use of immunochemistry allowed histological classification in 24 of 42 cases initially diagnosed as poorly differentiated cancer at morphology alone (57.1%), of which 15 of 25 of initial unclassified NSCLCs (60%).

The 19 diagnostic biopsies with result of specific benign disease included 10 cases of infection (52.6%), 8 cases of non-infectious inflammatory disease (42.1%), and 1 case of benign tumor (5.3%).

### 3.3. Comparison of Diagnostic and Nondiagnostic Biopsy Results

As shown in Table 2, between the biopsies with diagnostic results and those with nondiagnostic results there were statistically significant differences in terms of presence or absence of emphysema, lesion size, number of samples, indications to biopsy, and final diagnosis (*p* values < 0.05).

The presence of emphysema on CT images was significantly associated with nondiagnostic biopsy results (*p* = 0.026). The mean lesion size was also significantly different between the two groups (*p* = 0.039) and the proportion of lesions with diameter ≤ 20 mm resulted significantly higher in nondiagnostic cases compared to diagnostic cases (27.2% vs. 13.3%; *p* = 0.047).

There were no statistically significant differences between diagnostic and nondiagnostic groups in other demographic data including age, sex and history of cancer, and lesion characteristics including CT attenuation, presence or absence of spiculations and location. In particular, nondiagnostic biopsy results were obtained in 4 of 16 partially solid lesions (25%) and in 29 of 167 solid lesions (17.4%), however the nondiagnostic rate was not significantly higher for partially solid nodules compared to solid nodules.

With regard to biopsy-related factors, the number of samples was significantly associated with diagnostic biopsy results (*p* = 0.017), whereas there were no statistically significant differences between diagnostic and nondiagnostic groups in needle path length, needle size and repetition or not of biopsy during the same session. The occurrence of complications was also not significantly associated with biopsy results.

A final diagnosis of benign lesion was significantly associated with nondiagnostic biopsy results (*p* = 0.001) and, in accordance with this finding, more nondiagnostic results were found when the indication to biopsy was the diagnosis of undetermined pulmonary lesions in order to differentiate benign from malignant diseases (*p* = 0.002).

Results of the multivariate analyses for independent risk factors of nondiagnostic biopsy results are shown in Table 3. The significant independent risk factors were lesion size ≤ 20 mm (*p* = 0.006) and final diagnosis of benign disease (*p* = 0.001).

## 4. Discussion

In this study, we analyzed pathological results from 183 CT-guided lung biopsies to differentiate diagnostic from nondiagnostic cases and identify the factors affecting biopsy results. Our results showed that CT-guided lung biopsy was capable to provide a specific diagnosis in 150 of 183 cases (82%), resulting in an acceptable low nondiagnostic rate of 18%. Most biopsy specimens with malignant diagnoses were suitable for specific tumor subtyping (86%), particularly in NSCLCs.

Finally, small lesion size and final benign diagnosis were independent risk factors for nondiagnostic results of lung biopsy.

Establishing a specific diagnosis either of malignancy or benign disease with biopsy can have a direct impact on clinical decision making. A diagnostic procedure resulting in a specific benign lesion often allows the patient to avoid thoracic surgery, which is more invasive and associated with higher costs. In the same way, when a malignant lesion is rendered by biopsy, the diagnosis can be considered conclusive because of the extremely low false-positive rates [13].

Lung biopsy samples with no specific findings are not uncommon in clinical practice and represent a diagnostic challenge due to uncertainty on their clinical value. To our knowledge, only recently some studies have focused on nondiagnostic results from CT-guided lung biopsies. Some authors reported nondiagnostic rates ranging from 15% to 22% [19,21,24,25], whereas Lee et al. [20], in a multicenter study examining the malignant risk of nondiagnostic biopsies, found a percentage of nondiagnostic cases of 28%.

We calculated a nondiagnostic rate of 18%, which is in line with most prior results. However, the unavailability of an onsite pathologist during biopsy procedures in the present study may have affected the rate of nondiagnostic cases. Indeed, immediate feedback on incomplete specimens can help reduce the incidence of nondiagnostic biopsy results.

In our analysis conducted on 33 nondiagnostic biopsies, 22 cases were finally diagnosed as malignant, resulting in a high false negative rate (67%) and a negative predictive value of only 33%. Compared to our results, prior studies reported higher negative predictive values for nondiagnostic cases, ranging from 51% to 68% [21,26]. This difference may be due to their categorization of the uncertain biopsy results into nonspecific benign and nondiagnostic groups, that did not specifically include the cases with atypical cells. As demonstrated by Lee et al [20], biopsy specimens categorized as atypical cell lesions are associated with a high likelihood of malignancy (90%). In agreement with these results, we found that all nondiagnostic biopsies containing atypical cells had a final diagnosis of malignancy (100%).

Another possible explanation of the discrepancy reported above may be the high prevalence rate of cancers observed in our total series of 183 biopsies (87%), with a consequent increase in malignancy rate among nondiagnostic cases.

Furthermore, nearly half of missed cancers among nondiagnostic biopsies were the result of an insufficient tissue sampling, which may explain our high proportion of false negative cases for malignancy.

Based on our experience, lesions resulting in nonspecific diagnosis or insufficient material at biopsy should be further investigated with re-biopsy, surgical resection or observed with a close follow up since a substantial, although variable, number of these cases may be finally malignant. In patients with these uncertain biopsy results, the integration between radiological, clinical and pathological data is crucial for driving management decisions and making the correct diagnosis [24]. In our institution, nondiagnostic cases of lung biopsy are generally subjected to a multidisciplinary evaluation in order to identify all available information on the individual patient and decide the adequate diagnostic workup. Discordant lesions in which a nondiagnostic biopsy result occurs in a setting of high clinical-radiological suspicion of malignancy are an indication to further biopsy or surgery.

It is important to specify that, among nondiagnostic biopsies, those with nonspecific benign findings such as normal structure, fibrous tissue or generic inflammation, have a high chance to be a truly benign lesion, as recently demonstrated [27]. Similarly, we found that most of the cases with nonspecific benign results at biopsy were finally determined to be benign lesions (62%).

In our study, a specific diagnosis of malignancy or benign disease was made in 150 biopsies, of which 131 were indicative of malignancy (87%) and 19 of benignity (13%). It should be noted that all the 131 lesions with malignant results at biopsy had a final diagnosis of malignancy, with no occurrence of false positive cases. In accordance with previous studies also reporting a very low false positive rate [10,11,12,13], this result confirms that a lung biopsy with a pathological result of malignancy is reliable. On the other hand, lung biopsy is less effective in ruling out cancer. In the present study, we found six missed cancers among the lesions with diagnostic biopsy results. This finding is not perfectly comparable with those of previous reports focusing on the diagnostic yield of CT-guided lung biopsy, since the definition of diagnostic and nondiagnostic results varies in the different studies [10,11,12,14,15,16,17]. In our dataset, of the six cancers with an initial diagnosis of benignity at biopsy, two cases were classified as inflammatory disease and four cases as infection, probably because of the coexistence of these benign alterations and a malignant process in the same lesion. In particular, we reported three cases of lung cancer incorrectly diagnosed as bacterial pneumonia at biopsy due to the presence of a large consolidative component representing the parenchymal infection secondary to bronchial obstruction by tumor tissue. Large lesion size and difficulty in the differentiation of tumor from post obstructive consolidation may have contributed to sampling error and incorrect diagnosis of these lesions. Furthermore, we observed that the rate of specific benign biopsy results among the lesions with a definitive diagnosis of benignity was 54%, which falls in the wide range of results reported in previous studies (17–82%) [26,28].

In the group of diagnostic biopsies, we evaluated the performance of CT-guided lung biopsy in the histological classification of lung cancer, particularly of NSCLC. As known [9], a precise tumor definition in NSCLC has a great clinical relevance due to the differences in medical treatment between squamous and non-squamous tumors. For this purpose, the use of immunohistochemistry in addition to morphology has been demonstrated to reduce the proportion of unclassified NSCLCs [29].

In our study, among the 103 lesions with biopsy results of NSCLC, 93 cases (90%) were specifically subtyped and only 10 cases (10%) were diagnosed as NSCLC—NOS. In line with the recent evidence [29], we also found that with immunohistochemistry the rate of NSCLC-NOS decreased from 24% to 10%. Indeed, of 103 NSCLCs 25 cases without typical morphological characteristics of squamous cell cancer or adenocarcinoma were initially classified as poorly differentiated cancers, while after evaluation by immunohistochemistry only 10 cases remained unclassified.

By multivariate analysis, we identified which variables related to study population, lesions and biopsy procedure were associated to an increased risk of nondiagnostic biopsy.

As recently demonstrated by Lee et al. [20] and Tipaldi et al. [25], the nondiagnostic rate of lung biopsies tends to be higher with small lesions. Other authors also reported that the diagnostic accuracy of lung biopsy declines as the lesion size gets smaller, especially with a diameter ≤ 20 mm [10,11]. Based on these results, we established 20 mm as a threshold for analysis of differences between diagnostic and nondiagnostic biopsies.

Similar to previous literature, in the present study lesion size significantly affected biopsy outcome. In particular, a diameter of 20 mm or smaller was determined to be an independent risk factor for nondiagnostic biopsy results.

Prior studies demonstrated that the final diagnosis of benignity is a risk factor for biopsy failure [19,30]. Our findings were consistent with these results. About half of the benign lesions had nondiagnostic results at biopsy, whereas only 14% of the malignant lesions had nondiagnostic biopsy results, with a statistically significant difference between the two groups. Furthermore, the final diagnosis of benign disease was an independent predictor of nondiagnostic biopsy, proved to be stronger than small lesion size.

Our study had some limitations. One limitation is that this study was retrospective. Data was obtained mainly from biopsy reports or directly from CT images of the procedure and some of them were incompletely documented or not recorded. Furthermore, our analysis included CT-guided lung biopsies performed over a relatively long period of time. Reporting templates have changed, with possible slight variations in how data were reported. As described above, the indications and some technical aspects of biopsy have also evolved over time. However, it should be considered that all biopsies were performed by the same experienced radiologist, thus making the procedure more standardized. Lastly, in the present study, we did not analyze the biopsy data regarding molecular tumor analysis. Recently, the identification of specific mutations in patients with advanced non-squamous NSCLC has become essential to select those more likely to respond to targeted therapies. Considering its importance and complexity, we believe that this issue should be the object of future extensive and dedicated studies.

## 5. Conclusions

The present study demonstrated that CT-guided transthoracic biopsy is an effective method for providing a specific diagnosis of pulmonary lesions, which in our analysis was obtained in 82% of the cases. Based on our results, lung biopsy, if positive for malignancy, is reliable for diagnosis of cancer and shows an excellent performance in histological classification of NSCLC, whereas, in the case of specific benign findings, the procedure is less valuable for excluding cancer. We had a relatively low proportion of nondiagnostic cases (18%), in which the diagnosis remained uncertain. In these patients, the likelihood of malignancy may be significant, thus further diagnostic investigations or a close follow-up are generally required. Finally, lesion size ≤ 20 mm and final diagnosis of benign disease were independent risk factors for nondiagnostic biopsy results.

## Figures and Tables

**Table 1:** Characteristics of patients, lesions and biopsy procedure.

Patient Characteristics	
Age (y)	68.1 ± 10.5 (15–89)
Sex male female	120 (70.6) 50 (29.4)
Emphysema yes no	72 (42.4) 98 (57.6)
Smoker yes no	92 (54.1) 78 (45.9)
History of cancer yes no	36 (21.2) 134 (78.8)
**Lesion characteristics**	
Size (mm)	35.9 ± 17.2 (11–91)
CT attenuation solid partially solid	167 (91.3) 16 (8.7)
Spiculations yes no	49 (26.8) 134 (73.2)
Location Upper or middle lobes Lower lobes	109 (59.6) 74 (40.4)
**Biopsy characteristics**	
Indication to lung biopsy Diagnosis of undetermined lesions Tumor classification and/or molecular analysis	63 (34.4) 120 (65.6)
Needle size 18 gauge 21 gauge	110 (60.1) 73 (39.9)
Needle path length (mm)	22.3 ± 13.8 (0–63)
Number of samples	2.9 ± 1.0 (1–5)
Repetition of biopsy for single session yes no	9 (4.9) 174 (95.1)
Major complications Minor complications Pneumothorax Parenchymal bleeding Hemoptysis	3 (16.4) 66 (36.1) 33 (18.0) 29 (15.9) 4 (2.2)

Note: Data are presented as mean ± standard deviation (range) or numbers (%).

**Table 2:** Comparison between biopsies with diagnostic and nondiagnostic results.

Variable	Diagnostic Results (*n* = 150)	Non Diagnostic Results (*n* = 33)	*p*-Value
**Patients**			
Age (y)	68.1 ± 10.2	67.9 ± 12.0	0.911
Sex (male/female)	104/46	26/7	0.278
Emphysema (yes/no)	59/91	20/13	0.026
Smoker (yes/no)	85/65	15/18	0.241
History of cancer	31/119	8/25	0.650
**Lesion characteristics**			
Size (mm)	30.6 ± 15.7	37.1 ± 17.4	0.039
Size range (≤20 mm/>20 mm)	20/130	9/24	0.047
CT attenuation (solid/partially solid)	138/12	29/4	0.448
Spiculations (yes/no)	38/112	11/22	0.347
Location (upper or middle lobes/lower lobes)	91/59	18/15	0.517
**Biopsy characteristics**			
Indication to lung biopsy Diagnosis of undetermined lesions Tumor classification and/or molecular analysis	44 106	19 14	0.002
Needle size (18 gauge/21 gauge)	88/62	22/11	0.395
Needle path length (mm)	21.6 ± 13.5	25.3 ± 15.3	0.208
Number of samples	3.0 ± 1.0	2.6 ± 0.9	0.017
Repetition of biopsy for single session (yes/no)	6/144	3/30	0.207
Complications (yes/no)	55/95	14/19	0.537
Final diagnosis (benign/malignant)	13/137	11/22	0.001

Note: Data are presented as mean ± standard deviation or numbers.

**Table 3:** Independent risk factors for nondiagnostic biopsy results at multivariate analysis.

Variable	OR Value	95% CI	*p*-Value
Lesion size ≤ 20 mm	1.569	1.370–1.796	0.006
Final benign diagnosis	3.442	2.991–4.070	0.001

OR, odds ratio; CI, confidence interval.

## Data Availability

The data presented in this study are available on request from the corresponding author. The data are not publicly available due to ethical reasons.
